# Study on the Flexural Performance of Hybrid-Reinforced Concrete Beams with a New Cathodic Protection System Subjected to Corrosion

**DOI:** 10.3390/ma13010234

**Published:** 2020-01-05

**Authors:** Yingwu Zhou, Yaowei Zheng, Lili Sui, Biao Hu, Xiaoxu Huang

**Affiliations:** Guangdong Provincial Key Laboratory of Durability for Marine Civil Engineering, Shenzhen University, Shenzhen 518060, China; ywzhou@szu.edu.cn (Y.Z.); zhengyaowei@cmhk.com (Y.Z.); suill@szu.edu.cn (L.S.); biaohu3-c@szu.edu.cn (B.H.)

**Keywords:** hybrid-reinforced concrete beam, flexural performance, impressed current cathodic protection, CFRP, SFCB

## Abstract

Steel corrosion is considered as the main factor for the insufficient durability of concrete structures, especially in the marine environment. In this paper, to further inhibit steel corrosion in a high chloride environment and take advantage of the dual-functional carbon fiber reinforced polymer (CFRP), the impressed current cathodic protection (ICCP) technique was applied to the hybrid-reinforced concrete beam with internally embedded CFRP bars and steel fiber reinforced polymer composite bar (SFCB) as the anode material while the steel bar was compelled to the cathode. The effect of the new ICCP system on the flexural performance of the hybrid-reinforced concrete beam subjected to corrosion was verified experimentally. First, the electricity-accelerated precorrosion test was performed for the steel bar in the hybrid-reinforced beams with a target corrosion ratio of 5%. Then, the dry–wet cycles corrosion was conducted and the ICCP system was activated simultaneously for the hybrid-reinforced concrete beam for 180 days. Finally, the three-point bending experiment was carried out for the hybrid-reinforced concrete beams. The steel bars were taken out from the concrete to quantitatively measure the corrosion ratio after flexural tests. Results showed that the further corrosion of steel bars could be inhibited effectively by the ICCP treatment with the CFRP bar and the SFCB as the anode. Additionally, the ICCP system showed an obvious effect on the flexural behavior of the hybrid-reinforced concrete beams: The crack load and ultimate load, as well as the stiffness, were enhanced notably compared with the beam without ICCP treatment. Compared with the SFCB anode, the ICCP system with the CFRP bar as the anode material was more effective for the hybrid-reinforced concrete beam to prevent the steel corrosion.

## 1. Introduction

In civil engineering, steel corrosion is considered as the main factor for the insufficient durability of concrete structures [1,2]. The bearing capacity of the structure is significantly decreased with the reduced steel section area and the weakened bonding strength, leading to the structural failure in advance and a huge economic loss. It is necessary to solve this intractable issue to extend structural service life, especially under the marine environment. As a kind of composite material, carbon fiber reinforced polymer (CFRP) possesses excellent properties, such as high tensile strength, good corrosion resistance, and is lightweight [3,4,5,6,7], making it an ideal alternative reinforcement to effectively prevent the steel corrosion. However, the FRP bar is brittle with low elastic modulus and high price, hindering the large-scale application in civil engineering [8,9].

To overcome the drawbacks of FRP bar and steel bar for concrete beams, a number of methods have been proposed, including composite bar and hybrid reinforcement system. Combining the advantages of steel bars and FRP materials, a new composite bar has been proposed recently: The steel fiber reinforced polymer composite bar (SFCB) with steel bar as the inner core, CFRP material as the outer layer, and resin as the bonding material [10,11]. Although the properties of SFCB have been verified experimentally including high elastic modulus and stable post-yield stiffness as well as excellent corrosive resistance, it has not yet been implemented completely in practical engineering. Additionally, due to the complexity of the manufacturing process, micropores and microcracks exist inevitably inside the composite bar, leading to the corrosion of the internal steel bar under adverse environments [12]. On the other hand, a hybrid reinforcement form for concrete structures has attracted lots of attention in the past few decades [13,14,15,16]. In the hybrid-reinforced concrete beams, the anticorrosive FRP bars are arranged in the corner area, which is more prone to corrosion, while the ductile steel bars are arranged inside the section. Based on the complementary advantages of two materials, such a hybrid reinforcement system is featured with improved ductility and enhanced bearing capacity compared with purely steel-reinforced concrete beams and FRP-reinforced concrete beams [15]. Many studies in the aspect of the flexural behavior for the hybrid-reinforced concrete beam have been conducted experimentally and numerically [13,17]. Although the hybrid reinforcement system is highly anticorrosive with enough ductility and promising application prospects, the internal steel bar is still vulnerable to corrosion in a high chloride environment. Other measures are required to further protect this system from corrosion in severe conditions.

Impressed current cathodic protection (ICCP) is an effective method to restrain steel corrosion [18,19,20]. In the ICCP system, the steel bar is connected to the negative pole of the direct current (DC) power supply, and the auxiliary anode material is connected to the positive pole. A current is applied to the steel bar to make the steel bar become a cathode and enough electrons are transferred to the surface of the steel bar, leading to the inhibition of the electron migration caused by steel corrosion. Therefore, the corrosion of the steel bar can be effectively avoided or lessened. It is well known that the successful application of ICCP largely depends on the selection of the appropriate anode material, including metalized zinc [21], conductive organic paints [22], activated titanium mesh [23], and coating-overlay anodes [24]. Unfortunately, these materials are either suffering from durability problems or are highly expensive, hindering the wide application of the ICCP technique in civil engineering. Owing to the excellent conductivity and electrochemical properties as well as corrosion resistance, CFRP is an ideal anode material and has been widely studied for the ICCP system in the last decade [25,26]. In the study of Sun et al. in 2016 [26], the corrosion behavior of CFRP laminate as the anodic material was focused with 3% NaCl solution, and the electrochemical performance as well as mechanical properties were identified. Recently, a dual function of CFRP in the ICCP system was demonstrated, i.e., externally bonded CFRP functioned as both the anode material for ICCP and strengthening material for structural strengthening system simultaneously [27,28,29,30,31]. However, to ensure the bonding reliability between the externally bonded CFRP and the concrete, as well as to improve the reinforcing effectiveness of the CFRP, developing an efficient conductive inorganic binder is urgent, which restricts the application of this system.

**Figure 1:**
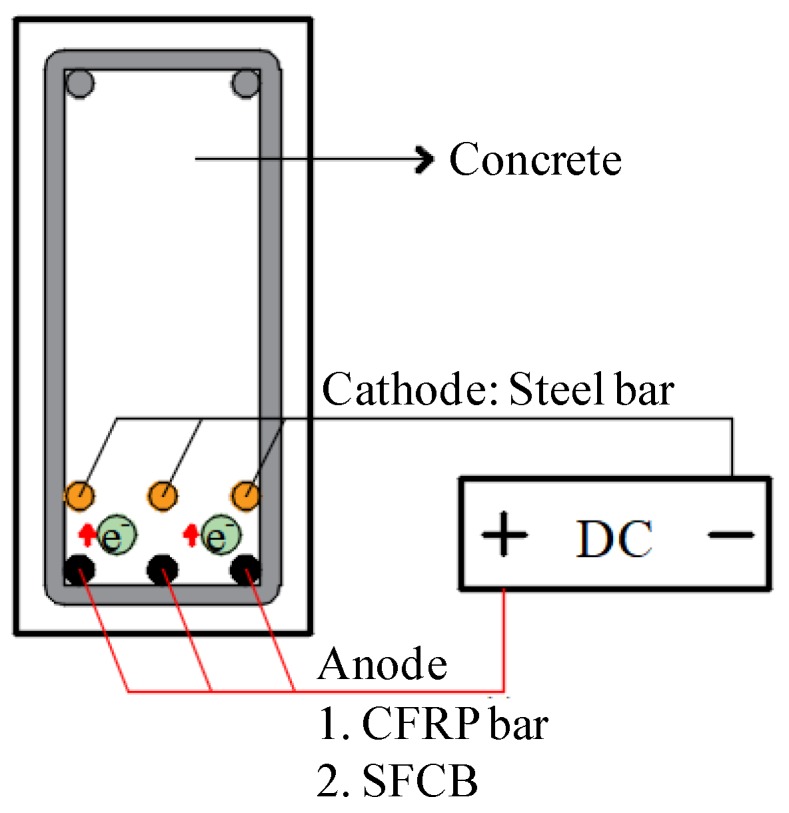
The cross-section of the hybrid-reinforced concrete beam with the new impressed current cathodic protection (ICCP) system.

**Figure 2:**
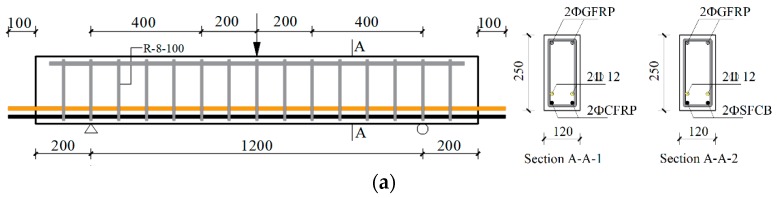

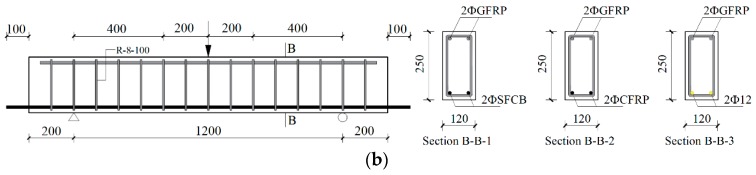
Experimental reinforced concrete (RC) beams (unit is mm): (**a**) Hybrid-reinforced, (**b**) single-reinforced.

Aiming at these issues, to further inhibit steel corrosion in a high chloride environment and take advantage of the dual-functional CFRP without the drawback of interface adhesive, the ICCP technique was applied to the hybrid-reinforced concrete (RC) beam with internally embedded CFRP bars and SFCB as the anode material in this paper, as shown in Figure 1 (the RC beam can be seen in Figure 2). In the hybrid-reinforced concrete beams, the anticorrosive CFRP bars and SFCBs were arranged in the lower layer while the ductile steel bars were arranged in the upper layer. The effect of the new ICCP system on the flexural performance of the hybrid-reinforced concrete beam subjected to corrosion was verified experimentally. First, to accelerate the dry–wet cycles corrosion in the whole process, an electricity-accelerated precorrosion test was performed for the steel bar in the hybrid-reinforced beams with a target corrosion ratio of 5%. Then, the dry–wet cycles corrosion was conducted and the ICCP system was activated simultaneously for the hybrid-reinforced concrete beam for 180 days. Finally, the three-point flexural experiment was carried out for the simply supported hybrid-reinforced concrete beams. After the tests, the steel bar was taken out from the concrete to quantitatively measure the steel corrosion. Results showed that the process of steel corrosion could be inhibited effectively by the ICCP treatment with the CFRP bar and the SFCB as the anode material. Additionally, the ICCP system showed an obvious effect on the flexural performance of the hybrid-reinforced concrete beams, especially for the CFRP anode. Compared with the SFCB anode, the ICCP system with the CFRP bar as the anode was more effective for the hybrid-reinforced concrete beam to prevent the steel corrosion. In general, the key issue of this paper was to investigate the flexural performance of hybrid RC beams with CFRP bar or SFCB as the anode material in the ICCP system subjected to corrosion, leading to the differences in CFRP type and test contents compared with other references [26,28]. Furthermore, considering the sea sand and seawater utilized and dry–wet cycle corrosion performed in this experiment, it can be inferred that the new ICCP system is a promising method to protect the steel bar subjected to the marine environment, and can further promote the utilization of sea sand and seawater in civil engineering.

## 2. Experimental Program

### 2.1. Test Specimens

As depicted in Figure 2, nine simply supported reinforced concrete beams were prepared, including 6 specimens for the hybrid-reinforced concrete beams and 3 specimens for the single-reinforced concrete beams. The dimension of the rectangular cross-section was 120 mm in width and 250 mm in height with a concrete cover thickness of 20 mm. For the hybrid-reinforced concrete beams, the CFRP bars and SFCBs were configured in the lower layer of the section, while the steels were arranged in the upper layer (i.e., 30 mm higher). For the single-reinforced concrete beams, the material of the reinforcement was selected as steel, CFRP bar, and SFCB, respectively. The erection and stirrup were selected as glass fiber bars with a diameter of 8 mm.

In order to accelerate the dry–wet cycles corrosion, the electricity-accelerated precorrosion test was first performed for the steel bar in the 6 hybrid-reinforced concrete beams with a target corrosion ratio of 5%. After the surface concrete cracked at the level of upper steel, the beams were subjected to the dry–wet cycles corrosion with 5% NaCl solution and protected by the ICCP system simultaneously for 180 days. The detailed information of the experimental design is listed in Table 1. The specimen is named with the following principle: UP means that the ICCP system was not activated; CP means that the ICCP system was activated; RE means without dry–wet cycles corrosion; S represents the controlled beams with single row reinforcements. For example, CFRP-CP is the hybrid-reinforced concrete beam (steel for the upper layer and CFRP bars for the lower layer) with the activated ICCP system under the dry–wet cycles environment.

### 2.2. Material Properties

#### 2.2.1. Reinforcements

**Figure 3:**
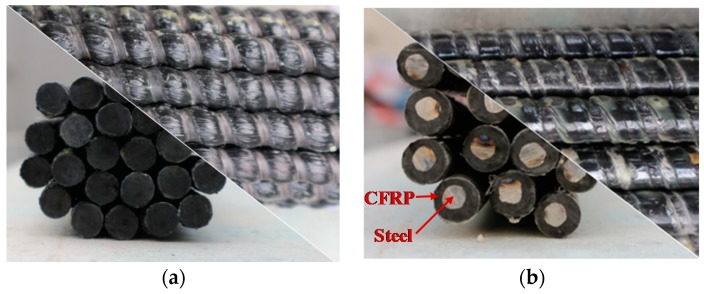
Experimental reinforcement: (**a**) carbon fiber reinforced polymer (CFRP) bar, (**b**) steel fiber reinforced polymer composite bar (SFCB).

As shown in Figure 3, the CFRP bar and SFCB used in this paper were produced by Jiangsu Nantong Intelligent Fiber Technology Company (Nantong, China). The carbon fiber was provided by Toray T700 carbon fiber (Toray, Tokyo, Japan) with a volume fraction of approximately 60%. To show a better conductive performance for CFRP, the resin layer on the surface of CFRP was removed before the test. The diameter of the SFCB was 12 mm, with the diameter of the inner steel bar equal to 8 mm, and the thickness of the outer carbon fiber was 2 mm. Additionally, the ribbed steel bar (HRB400) with a diameter of 12 mm was utilized in this study. The mechanical properties of the reinforcements are given in Table 2 (mean value of three identical specimens).

#### 2.2.2. Concrete

The mix proportion of concrete in weight was Mc:Mw:Ms:Mg (i.e., cement:water:sand:gravel) = 1:0.49:1.58:2.81. Ordinary Portland cement was utilized, and the natural aggregates with a diameter ranging from 12 mm to 20 mm were selected as the gravel. To simulate the chloride contaminated RC structures, sea sand and seawater were utilized in this experiment with the chloride ion content of 0.05% and 2.0 × 10^4^ mg/L, respectively. The seawater was taken from the offshore of Fuyong Wharf in Shenzhen with the corresponding ionic parameters shown in Table 3. The concrete compressive strength was verified by the experimental test for three cubic concrete specimens (100 mm × 100 mm × 100 mm) after curing 28 days. The average compressive strength was obtained at 42.37 MPa.

### 2.3. Constant Current Accelerated Corrosion Test

#### 2.3.1. Corrosion Time

In the natural environment, the corrosion of the steel reinforcement inside the concrete beam is actually a very long process. Considering the time factor and the limited experimental conditions, precracking was performed for the hybrid-reinforced concrete beam to accelerate the following corrosion of the steel in the dry–wet cycles environment. The concrete on the surface of the steel expanded and cracked by the corrosion product, leading to the fact that oxygen, water, and other harmful ions are more likely to enter concrete and react with steels.

According to Faraday’s law, in an electrolytic reaction, the mass consumed or produced by the reactants is proportional to the conduction time and the current intensity. The mass loss of steel due to the impressed current can be evaluated as [32,33]:

(1) $$\Delta m=\frac{MIt}{ZF}$$

in which *M* represents the molar mass of iron (56 g/mol), *Z* denotes the number of electrons that iron atoms lose by generating Fe^2+^, *F* is the Faraday constant (96,487 c·mol^−1^), *t* is the conduction time(s), and *I* is the current intensity (A) which can be expressed as:

(2) I=is=iπdl

where *i* means the electric current density (μA/cm^2^), *d* is the steel diameter (cm), and *l* is the length of the corroded steel bar (cm).

The corrosion ratio *η* can be measured by the ratio of steel mass loss to the mass before corrosion, i.e.,

(3) $$\eta =\frac{\Delta m}{m}\times 100\%$$

In this experiment, the current density was set to 400 μA/cm^2^. Based on Equation (2), the current intensity evaluated was 0.48 A. Thus, a DC power supply with an output DC voltage of 0–60 V and a current of 0–3 A was selected. The average density of steel was 7.37 g/cm^3^. Thus, to obtain the target corrosion ratio of 5%, 264.5 h were required for the electric precorrosion.

#### 2.3.2. Electric Precorrosion Experiment

**Figure 4:**
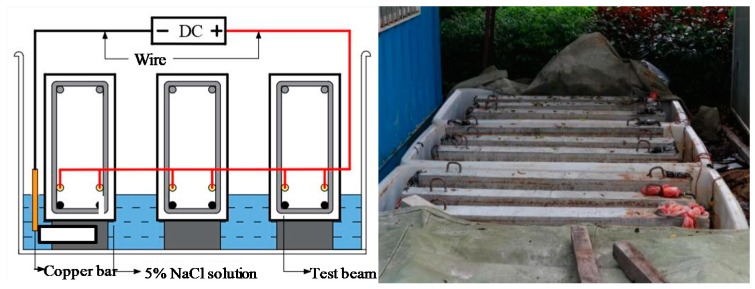
Setup of the electric corrosion experiment.

**Figure 5:**
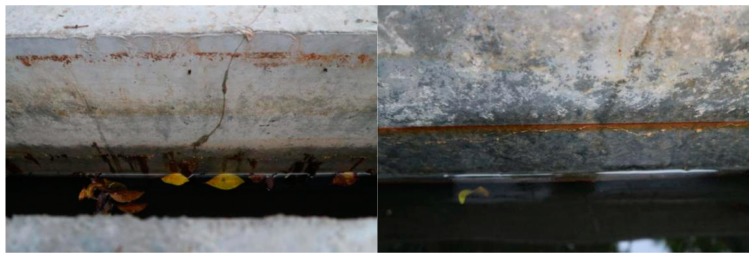
Cracks along the steel bar after the precorrosion.

After the curing for 28 days, the electric corrosion experiment was conducted on the hybrid-reinforced concrete beam. As shown in Figure 4, three specimens were placed in the 5% NaCl solution, and wires were drawn out: The steel bar was connected to the positive pole of the DC power supply and the cooper bar was connected to the negative pole. The three specimens were combined in series, while the two upper tensile steel bars of each specimen were connected in parallel. The liquid level was lower than the upper corroded steel bars and marked by a sign. To avoid the excessive current consumption in the process of demineralization and a large discrepancy between the actual corrosion result and the evaluated one, the chloride ion was allowed to permeate into the concrete layer in advance to destroy the passivation film on the steel surface. Therefore, the specimens were soaked in the solution for 72 h firstly, and then the circuit was electrified according to the current intensity and duration (the voltage applied was in the range of 2 V to 3 V). During the experiment, the output current was checked regularly to prevent short-circuit, and water was added in time to ensure the constant concentration. As the electric time increased, the corrosion products at the bottom of the test box also increased: Red rust (Fe_2_O_3_) and black rust (Fe_3_O_4_) produced by incomplete oxidation. After the electric precorrosion, it was found that cracks appeared along with the longitudinal steel with corrosion products, as shown in Figure 5.

### 2.4. Dry–Wet Cycles Accelerate Corrosion

**Figure 6:**
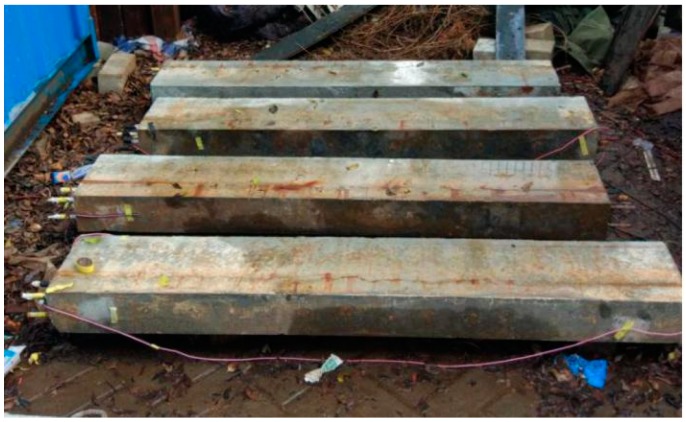
Test specimens subjected to the dry–wet cycles corrosion.

As a simulation of the tidal action for seawater, the dry–wet cycles can reflect the real corrosion environment, and the corrosion state of steel bars can be identified. Due to the limitation of lab conditions and specimens, the outdoor dry–wet alternate environment was utilized here: The concrete specimen was fully infiltrated in 5% NaCl solution every morning and evening, and then dried by natural air. As shown in Figure 6, the experiment was located outside the school of civil engineering at Shenzhen University, and the corrosion lasted for 180 days.

### 2.5. New ICCP System Experiment

**Figure 7:**
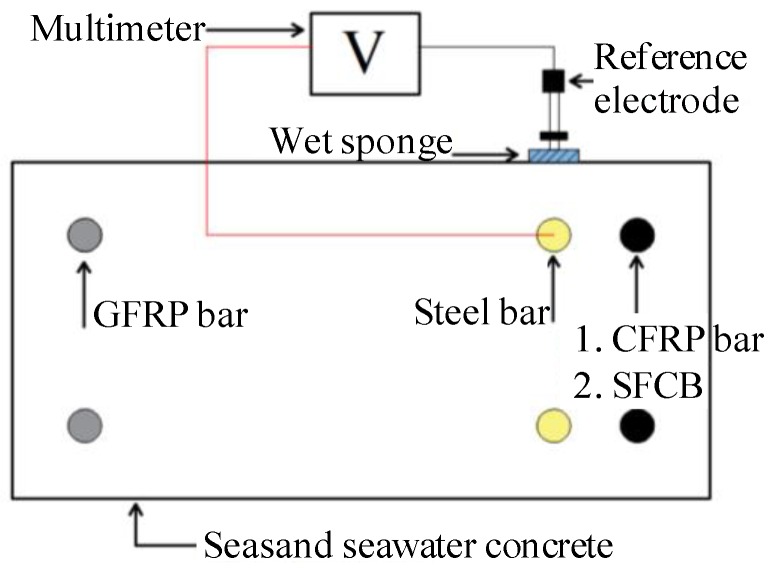
Schematic diagram of the potential measurement.

After the start of the dry–wet cycles corrosion, the ICCP treatment was performed for the specimen. As shown in Figure 1, the CFRP bar or SFCB in the lower layer was connected to the positive pole, while the steel bar in the upper layer was connected to the negative pole. The self-corrosion potential of the steel bar was measured twice a week, as shown in Figure 7. The protection current density was set to 198 mA/m^2^ and 248 mA/m^2^ for CFRP bars and SFCB, respectively.

### 2.6. Three-Point Bending Test

**Figure 8:**
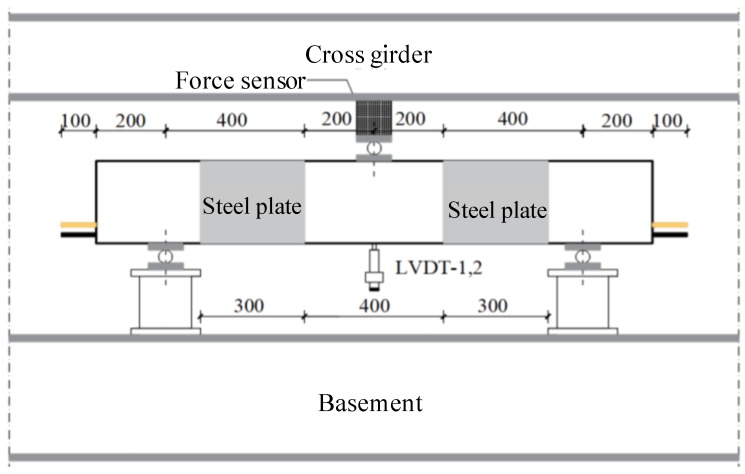
Setup of three-point bending test.

As depicted in Figure 8, the three-point bending test was conducted for all the concrete beams with a 10,000 KN hydraulic long column testing machine (Hengle, Jinan, China). Displacement control was selected, and the loading rate was 0.5 mm/min. We kept loading until the following situations occurred: (1) Concrete crush, or (2) reinforcement rapture, or (3) shear failure. To avoid the shear failure mode for the hybrid-reinforced concrete beam, the steel plate was used to improve the shear capacity.

In this experiment, the Dewsoft dynamic data acquisition box was utilized to collect the data of force sensor, displacement sensor, and strain gauge with a frequency of 1 Hz. The measuring content is described as follows: (1) Load measurement: A force sensor with a range of 475 kN was placed on the steel pad to measure the applied load; (2) deflection measurement: Two displacement sensors with a range of 50 mm were placed on the mid-span of the test beam to measure the mid-span vertical displacement; and (3) crack measurement: A fracture tester was utilized to measure the maximum crack width in the curved shear section.

### 2.7. Derusting and Weighing of the Steel Bar

After the bending test, the steel bars in the specimen were taken out to inspect and measure the corrosion degree quantitatively. According to the method recommended in American Society of Testing Materials (ASTM) G1-03 [34], the corrosion products on the steel bar were removed within 25 min. Then, the steel bar was cleaned with absolute ethyl alcohol immediately and weighed after it dried. Finally, the length of the steel bar was measured with the Vernier caliper to evaluate the line density, i.e., the ratio of the weight of the steel bar to the length.

## 3. Results and Discussions

### 3.1. Electrochemical Protection Effect

**Figure 9:**
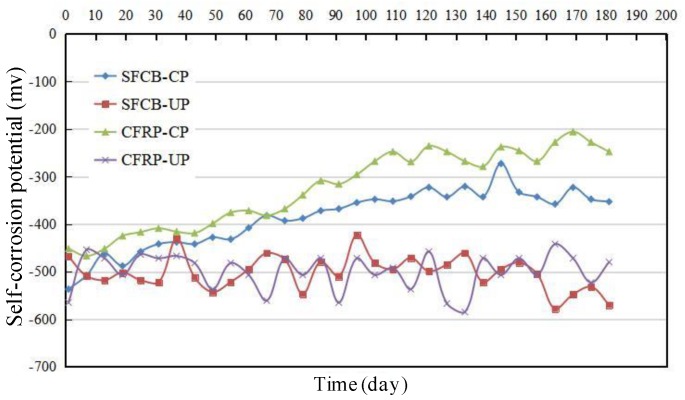
Self-corrosion potential for the steel bar in the dry–wet cycles experiment.

Figure 9 presented the self-corrosion potential of the steel bar in the dry–wet cycles experiment with the new ICCP system. It can be observed that the self-corrosion potential of the steel bar was quite negative (about −500 mV) after the precorrosion. As time went on, the self-corrosion potential gradually developed in a positive direction for the test beams with ICCP treatment (i.e., CFRP-CP and SFCB-CP) while keeping in a quite negative level for the test beams without ICCP treatment (i.e., CFRP-UP and SFCB-UP). The self-corrosion potential of CFRP-CP was notably higher than that of SFCB-CP after 70 days. The final potential after 180 days was −230 mV and −350 mV for CFRP-CP and SFCB-CP, respectively. Therefore, compared with SFCB anode, the cathodic protection effect was more obvious for the anode of the CFRP bar. The variation of the steel interruption potential for each specimen is listed in Table 4. It can be inferred that the polarization attenuation values were larger than 100 mV, which satisfies the criterion of the minimum polarization attenuation of cathodic protection [35]. Therefore, it is possible to utilize the CFRP bar and SFCB as the anode material in the ICCP system to inhibit the steel corrosion in a severe environment.

### 3.2. Loading Capacity Analysis

#### 3.2.1. Failure Modes

**Figure 10:**
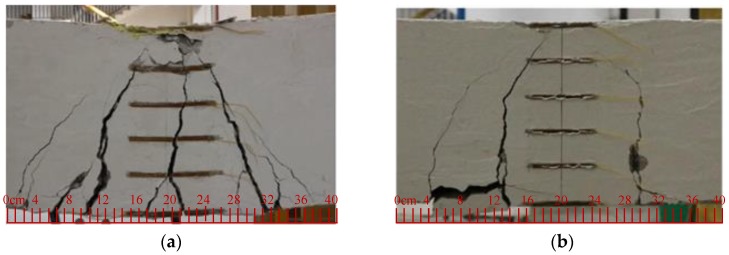

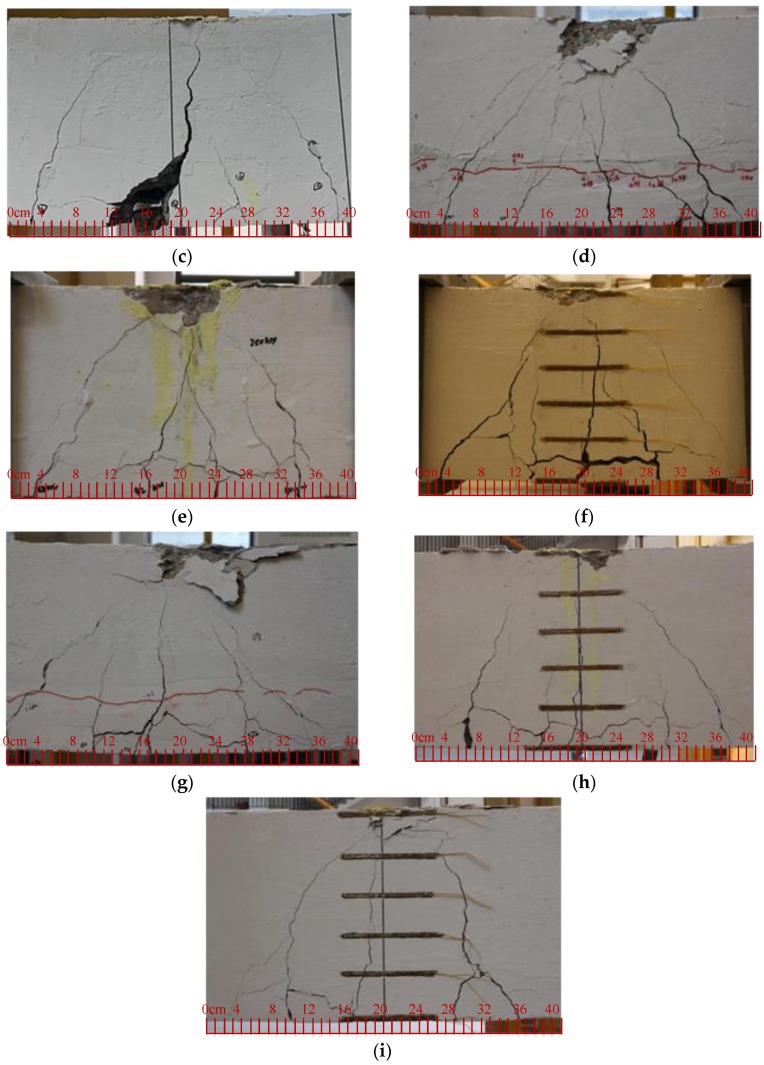
Failure modes of the test beams: (**a**) Steel-S, (**b**) SFCB-S, (**c**) CFRP-S, (**d**) SFCB-RE, (**e**) SFCB-UP, (**f**) SFCB-CP, (**g**) CFRP-RE, (**h**) CFRP-UP, (**i**) CFRP-CP.

In this three-point bending experiment, the specimens were tested to failure, and the observed failure modes included concrete crushing and reinforcement rupture, as shown in Figure 10. For the Steel-S specimen, concrete crushed in the compression zone, indicating the loss of bearing capacity. For the SFCB-S specimen, the core steel bar yielded first and then the fiber on the surface ruptured as the load continued to increase. For the CFRP-S specimen, failure occurred suddenly with a brittle rupture of CFRP bar. For the hybrid-reinforced concrete beams, failure occurred when the concrete crushed in the compression zone.

#### 3.2.2. Load-Deflection Curves

**Figure 11:**
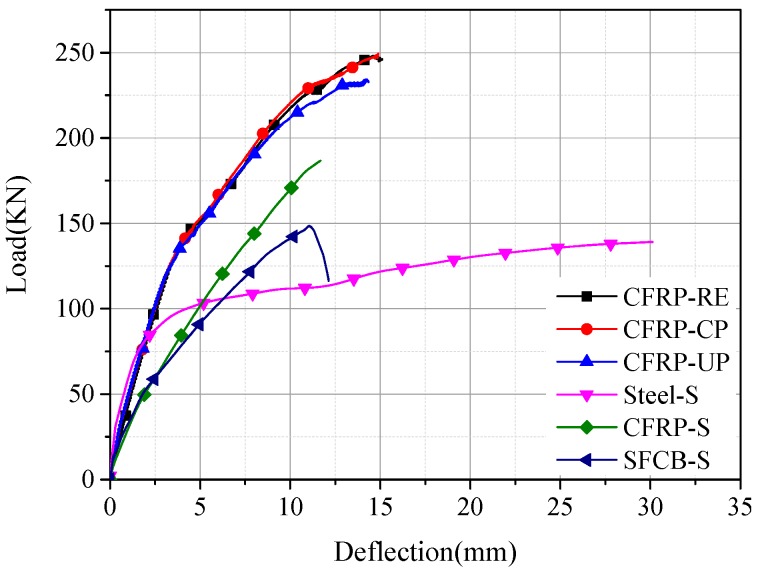
Load-deflection curves for the hybrid-reinforced beams with CFRP bar.

**Figure 12:**
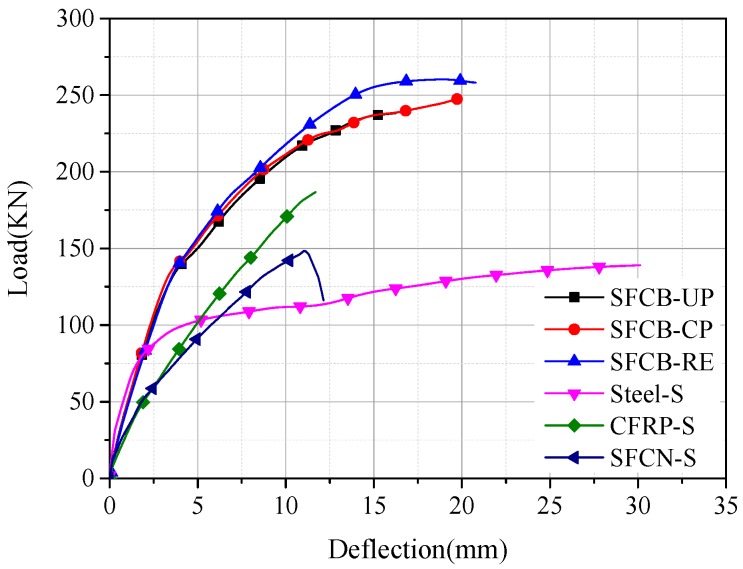
Load-deflection curves for the hybrid-reinforced beams with SFCB.

The load-deflection curves for the RC beams configured with SFCB and CFRP bars are plotted in Figure 11 and Figure 12, respectively. It has to be mentioned that the load-deflection curves for CFRP-CP, CFRP-UP, SFCB-CP, and SFCB-UP are related to the duration of the dry–wet cycles corrosion. Before cracking, the load-deflection curve increased linearly, and the stiffness for the single-reinforced concrete beam was obviously smaller than that of the hybrid-reinforced concrete beam. After cracking, there was a slight decrease in the stiffness for all the beams. As the load continued to increase, the core steel inside the SFCB first yielded, and then the tensile load was sheared by the externally wrapped CFRP, leading to the post-yield stiffness for SFCB. The final ultimate capacities of CFRP-S and SFCB-S are namely 34.28% and 6.74%, larger than that of Steel-S.

After cracking, the variation tendency of the stiffness was the same for the hybrid-reinforced concrete beams with CFRP bar. As the load continued to increase, the stiffness of CFRP-CP was close to that of CFRP-RE, indicating that the stiffness did not change obviously for the beam with ICCP treatment. Besides, CFRP-UP showed the smallest stiffness, indicating that there existed a decrease in the stiffness for the beam without cathodic protection.

After the yielding of the steel bar, the stiffness of SFCB-CP was slightly larger than that of SFCB-UP, since the corrosion was less serious for the upper steel bar in SFCB-CP with cathodic protection. As the load continues to increase, a distinguished difference appeared for the stiffness of the three beams: The value of SFCB-RE was obviously larger than that of SFCB-CP and SFCB-UP, indicating that the effectiveness of the ICCP system with SFCB as the anode material was not significant.

#### 3.2.3. Critical Loads

The load capacity for each specimen is analyzed in detail in the following part. The values of critical loads, including cracking load, yield load, and ultimate load, for each specimen are listed in Table 5.

(1) Cracking Load

For the concrete beams configured with single reinforcement, CFRP-S and Steel-S showed the smallest and largest cracking load capacity, respectively, i.e., 14 kN and 27.9 kN. Assuming that the steel ratio was 100% and 0% for Steel-S and CFRP-S, respectively, then the steel ratio for SFCB was 25%. Thus, it can be inferred that the cracking load increased as the steel ratio increased. Additionally, compared with the single-reinforced concrete beam, the cracking load was much larger for the beam with the hybrid reinforcement. For the hybrid-reinforced beams with CFRP bar, compared with the reference beam without suffering dry–wet cycles corrosion (i.e., CFRP-RE), the cracking load declined by 17% and 4.3% for CFRP-UP (i.e., without ICCP treatment) and CFRP-CP (i.e., with ICCP treatment), respectively. It indicated that the new cathodic protection system with the CFRP bar as the anode was effective for controlling the initial cracking. For the hybrid-reinforced beams with SFCB, the cracking loads were very close.

(2) Yield Load

When the tension concrete was gradually out of work due to cracking, the load in the tension zone was shared by steel bars, CFRP bars, or SFCB. The steel yield existed for all the specimens except for the CFRP-S due to the brittle nature of the CFRP bar. For the single-reinforced concrete beam, since the steel diameter of Steel-S was larger than that of SFCB-S, the yield load was significantly larger for Steel-S (i.e., 99.03 kN for Steel-S and 50.86 kN for SFCB-S). For the hybrid-reinforced concrete beam, compared with the reference beam (i.e., CFRP-RE, SFCB-RE), the yield load of other hybrid-reinforced concrete beams decreased slightly. Additionally, the dry–wet cycles environment had a negative effect on the yield load. However, the effect of the ICCP treatment on the yield load was uncertain and not obvious.

(3) Ultimate Load

For the single-reinforced concrete beams, compared with Steel-S, the ultimate load had increased by 6.74% and 32.28% for SFCB-S and CFRP-S, respectively. Compared with the single-reinforced beams, the ultimate load was larger for the hybrid-reinforced beams. The ultimate capacity of CFRP-UP was 5.96% lower than that of CFRP-RE, indicating that corrosion occurred for the steel in the RC beam without ICCP treatment under the dry–wet cycles corrosion. Thus, the ultimate capacity decreased due to the reduced cross-section area of steel bars. The ultimate capacity of CFRP-CP was close to that of CFRP-RE, indicating that the new ICCP system with the CFRP as the anode was effective and had a positive effect on the ultimate load capacity. This phenomenon can be explained as follows: Electrons were transferred through the CFRP bar (anode) to the steel bar (cathode), making the steel bar in a state of noncorrosive or with a quite low corrosion rate. The cross-section area of the steel bar did not decrease, nor did the corresponding mechanical properties. Furthermore, the mechanical properties of the CFRP bar were basically unchanged without excessive consumption as the anode.

The ultimate capacities of SFCB-UP and SFCB-CP were 8.45% and 4.79% lower than that of SFCB-RE, respectively. It can be inferred that corrosion occurred for the steel without ICCP treatment under the dry–wet cycles corrosion, leading to a decrease of the ultimate capacity. However, the effect was not significant for the ICCP system with the SFCB as the anode. This can be explained as follows. According to the result of potential in Figure 9, the potential of the steel bar was in the immunity region. Thus, it can be inferred that there existed a deterioration of the carbon fiber on the surface of SFCB with ICCP treatment. Owing to this factor, the mechanical properties of SFCB were deteriorated, leading to a decrease in the load capacity of the concrete beam.

### 3.3. Crack Analysis

For the three-point bending test, cracks first occurred at the bottom of the beam in the loading area and then developed to the loading point. The distribution of cracks for the concrete beams can be observed in Figure 10. It can be found that (1) for the single-reinforced beams, the number of cracks decreased with the increase of the CFRP proportion in the reinforcement ratio, i.e., Steel-S and CFRP-S showed the largest and least number of cracks, respectively. This phenomenon can be explained by the fact that the beam configured with steel bars showed a better ductility. Additionally, due to the brittle failure mode for CFRP-S, the corresponding main crack width was the largest (see Figure 10c). (2) For the hybrid-reinforced beams, the number of cracks and the width was larger than that of SFCB-S and smaller than that of Steel-S. As described in Figure 5, due to the precorrosion treatment for the upper steel bar in the hybrid-reinforced concrete beam, transverse cracks appeared, which was different from the single-reinforced concrete beam. Compared with the hybrid RC beam configured with the CFRP bar, the beam configured with SFCB was featured with more cracks and larger width, owing to a higher steel ratio and a better ductility. Furthermore, there was no distinguished difference of crack number or the width between the beams with ICCP treatment and without ICCP treatment.

### 3.4. Line Density Loss of Steel Bar

**Figure 13:**
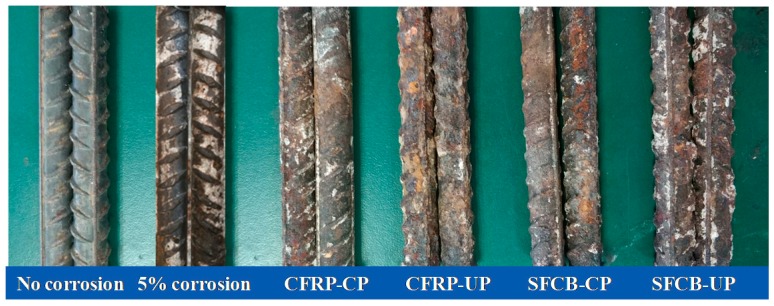
Morphology of the steel bar after the bending test.

After the bending test of the concrete beam, the steel bar was taken out, and the morphology is depictured in Figure 13. It is noted that the steel labeled with 5% corrosion refers to the precorrosion in Section 2.3.2. By visual inspection, it can be observed that, compared with the steel bar without corrosion, the surface of the steel bar in the specimen with 5% corrosion changed to a darker color. Compared with the steel bar with ICCP treatment (i.e., CFRP-CP and SFCB-CP), the rib transverse on the surface was less obvious and the corrosion was more serious for the steel bar without ICCP treatment (i.e., CFRP-UP and SFCB-UP), indicating that the ICCP treatment was conducive to inhibit steel corrosion with CFRP bar and SFCB as anode. Compared with the steel bar in the CFRP-CP specimen, the corrosion was more serious for the steel bar in the SFCB-CP specimen, indicating that the effect of the ICCP treatment on the inhabitation of steel corrosion was more obvious for the CFRP bar anode.

Besides the qualitative analysis of the steel corrosion based on the morphology, the corrosion degree was quantitatively measured by the loss of the line density of the steel bar in this study. The results are listed in Table 6. The mean value and the standard deviation (the value in the bracket) of the line density loss are given in the last column. Although the target precorrosion rate of the steel bar was set to be 5%, the actual line density loss of the steel bar was measured as 3.61%. The line density loss of the steel bar was 7.23% for CFRP-UP and SFCB-UP (i.e., much larger than that of 5% corrosion), indicating that the effect of the dry–wet cycles on the steel corrosion was notable. Compared with the line density loss for CFRP-UP, the value was evaluated as 4.22% for CFRP-CP (i.e., a little larger than that of 5% corrosion), indicating that the ICCP treatment was effective with CFRP bars as the anode and the corrosion process of steel bars was inhibited in the severe environment. However, compared with the line density loss for SFCB-UP, the value was evaluated as 6.63% for SFCB-CP, which is much larger than that of 5% corrosion. It can be inferred that the ICCP effectiveness was not obvious with SFCB as the anode, and the corrosion process of steel bars is suppressed partially in the severe environment. Therefore, the quantitative analysis of the corrosion can further verify that the corrosion inhibition effect was more obvious for the cathodic protection system with CFRP bars as the anode, which had a good agreement with the load capacity analysis.

## 4. Conclusions

In this paper, to further inhibit steel corrosion in a high chloride environment and take advantage of the dual-functional CFRP, the ICCP technique was applied to the hybrid-reinforced concrete beam with internally embedded CFRP bars and SFCBs as the anode material. In the hybrid-reinforced concrete beams, the anticorrosive CFRP bars and SFCBs were arranged in the corner area of stirrups where it is more prone to suffer from corrosion while the ductile steel bars were arranged above. The effect of the new ICCP system on the flexural behavior of the hybrid-reinforced concrete beam was verified experimentally in a severe environment. First, the electricity-accelerated precorrosion was performed for the steel bars in the hybrid-reinforced beams with a target corrosion ratio of 5%. Then, the dry–wet cycles corrosion was conducted and the ICCP system was activated for the hybrid-reinforced concrete beam for 180 days. Finally, the three-point flexural experiment was carried out for the hybrid-reinforced concrete beams. The steel bars were taken out from the concrete to quantitatively measure the corrosion ratio after flexural tests. Based on this study, the following conclusions can be drawn:

(1) With the CFRP bar and the SFCB as the anode material, the process of steel corrosion was inhibited effectively by the ICCP system. Compared with the steel bar without ICCP treatment, the self-corrosion potential of the steel bar was developing towards the corrosion immunity zone, and the loss of the line density was decreased. Furthermore, considering the sea sand and seawater utilized and the dry–wet cycle corrosion performed in this experiment, it can be inferred that the new ICCP system is a promising method to protect the steel bar subjected to the marine environment, and can further promote the utilization of sea sand and seawater in civil engineering.

(2) The ICCP system showed an obvious effect on the flexural performance of the hybrid-reinforced concrete beams. Compared with the hybrid RC beam without ICCP treatment, the crack load and ultimate load, as well as the stiffness, were enhanced notably. However, there was no distinguished difference of crack number and width between the beams with ICCP treatment and without ICCP treatment.

(3) Compared with the SFCB anode, the ICCP system with the CFRP bar as the anode material was more effective for the hybrid-reinforced concrete beam to prevent the steel corrosion. This can be confirmed by the result of the line density loss of the steel bar. There existed a deterioration of the carbon fiber on the surface of SFCB with ICCP, leading to the degradation in the mechanical properties of SFCB, which can be further verified by the result of self-corrosion potential and the ultimate load capacity.

## Figures and Tables

**Table 1:** Experimental design.

Specimen	Pre-Corrosion	Anode Material	ICCP	Dry–wet Cycles	Time (day)
CFRP-RE	5%	CFRP bar	N	N	0
CFRP-UP	5%	CFRP bar	N	Y	180
CFRP-CP	5%	CFRP bar	Y	Y	180
SFCB-RE	5%	SFCB	N	N	0
SFCB-UP	5%	SFCB	N	Y	180
SFCB-CP	5%	SFCB	Y	Y	180
SFCB-S	0%	-	N	N	N
CFRP-S	0%	-	N	N	N
Steel-S	0%	-	N	N	N

**Table 2:** Mechanical properties of the experimental reinforcements.

Type	*d* (mm)	*ε_y_* (%)	*f_y_* (MPa)	*ε_u_* (%)	*f_u_* (MPa)	*E*_I_ (GPa)	*E*_II_ (GPa)
CFRP bar	12	-	-	-	788.0	125.0	-
SFCB	12	0.23	515.6	0.48	716.1	224.2	146.2
Steel bar	12	0.21	425.1	-	572.8	201.4	-

Note: *d*, diameter; *ε_y_*, yield strain; *f_y_*, yield strength; *ε_u_*, ultimate strain; *f_u_*, ultimate strength; *E*_I_, elastic modulus; *E*_II_, post-yielding elastic modulus.

**Table 3:** Concentrations of main chemical ions in seawater (unit is mg/L).

Na^+^	K^+^	Ca^2+^	Mg^2+^	F^−^	Cl^−^	Br^-^	SO_4_^2−^	CO_3_^2−^
395	422.5	1.24 × 10^4^	1.0 × 10^3^	5.14	2.0 × 10^4^	48.55	1.82 × 10^3^	7.54

**Table 4:** Variation of the interruption potential for the concrete beams.

Specimen	Time (day)	Interruption Potential (mV)	Depolarization Potential (mV)	Attenuation (mV)
SFCB-CP	20	−705	−473	232
	60	−640	−401	239
	120	−502	−374	128
	180	−571	−368	203
CFRP-CP	20	−420	−248	172
	60	−444	−239	205
	120	−494	−234	260
	180	−426	−221	205

**Table 5:** The result of the load capacity for the test beams.

Specimen	Cracking Load (kN)	Yield Load (kN)	Ultimate Load (kN)	Yield Deflection (mm)	Ultimate Deflection (mm)
CFRP-RE	47.0	147.3	247.5	4.6	15.0
CFRP-UP	39.0	132.2	232.7	3.8	14.3
CFRP-CP	45.0	128.4	248.1	3.5	14.9
SFCB-RE	40.0	143.3	260.2	4.2	20.8
SFCB-UP	39.0	140.0	238.2	4.1	16.2
SFCB-CP	40.0	143.0	247.7	4.2	19.8
SFCB-S	15.4	50.9	148.5	1.9	11.3
CFRP-S	14.0	-	186.8	-	11.7
Steel-S	27.9	99.0	139.1	4.0	30.1

**Table 6:** Characterization of corrosion rate for steel bars.

Specimen	Weight (g)	Length (mm)	Line Density (g/mm)	Average Line Density (g/mm)	Line Density Loss
No corrosion	83.3	99.87	0.83	0.83	0% (0%)
	83.3	99.77	0.83		
5% corrosion	79.5	99.35	0.80	0.80	3.61% (0%)
	79.3	99.12	0.80		
CFRP-CP	78.8	99.23	0.79	0.795	4.22% (0.85%)
	78.8	98.86	0.80		
CFRP-UP	76.9	97.94	0.79	0.77	7.23% (3.40%)
	74.7	99.51	0.75		
SFCB-CP	76.8	99.16	0.77	0.775	6.63% (0.85%)
	76.2	97.51	0.78		
SFCB-UP	76.4	100.58	0.76	0.77	7.23% (1.70%)
	74.7	95.7	0.78		
